# Spatial organization and proteome of a dual-species cyanobacterial biofilm alter among N_2_-fixing and non-fixing conditions

**DOI:** 10.1128/msystems.00302-23

**Published:** 2023-06-07

**Authors:** Mahir Bozan, Matthias Schmidt, Niculina Musat, Andreas Schmid, Lorenz Adrian, Katja Bühler

**Affiliations:** 1 Department of Solar Materials, Helmholtz-Centre for Environmental Research (UFZ), Leipzig, Germany; 2 Department of Isotope Biogeochemistry, Helmholtz-Centre for Environmental Research (UFZ), Leipzig, Germany; 3 Department of Environmental Biotechnology, Helmholtz-Centre for Environmental Research (UFZ), Leipzig, Germany; Colorado State University, Fort Collins, Colorado, USA

**Keywords:** *Tolypothrix*, biofilms, microbial interactions, CLSM, cyanobacteria, proteomics

## Abstract

Many disciplines have become increasingly interested in cyanobacteria, due to their ability to fix CO_2_ while using water and sunlight as electron and energy sources. Further, several species of cyanobacteria are also capable of fixing molecular nitrogen, making them independent of the addition of nitrate or ammonia. Thereby they hold huge potential as sustainable biocatalysts. Here, we look into a dual-species biofilm consisting of filamentous diazotrophic cyanobacteria *Tolypothrix* sp. PCC 7712 and heterotrophic bacteria *Pseudomonas taiwanensis* VLB 120 growing in a capillary biofilm reactor. Such systems have been reported to enable high cell densities continuous process operation. By combining confocal laser scanning and helium-ion microscopy with a proteomics approach, we examined these organisms’ interactions under two nitrogen-feeding strategies: N_2_-fixing and nitrate assimilation. Not only did *Pseudomonas* facilitate the biofilm formation by forming a carpet layer on the surface area but also did N_2_-fixing biofilms show greater attachment to the surface. *Pseudomonas* proteins related to surface and cell attachments were observed in N_2_-fixing biofilms in particular. Furthermore, co-localized biofilm cells displayed a resilient response to extra shear forces induced by segmented media/air flows. This study highlights the role of *Pseudomonas* in the initial attachment process, as well as the effects of different nitrogen-feeding strategies and operation regimes on biofilm composition and growth.

**IMPORTANCE:**

Cyanobacteria are highly interesting microorganisms due to their ability to synthesize sugars from CO_2_ while using water and sunlight as electron and energy sources. Further, many species are also capable of utilizing molecular nitrogen, making them independent of artificial fertilizers. In this study, such organisms are cultivated in a technical system, which enables them to attach to the reactor surface, and form three-dimensional structures termed biofilms. Biofilms achieve extraordinarily high cell densities. Furthermore, this growth format allows for continuous processing, both being essential features in biotechnological process development. Understanding biofilm growth and the influence technical settings and media composition have on biofilm maturation and stability are crucial for reaction and reactor design. These findings will help to open up these fascinating organisms for applications as sustainable, resource-efficient industrial workhorses.

## INTRODUCTION

Cyanobacteria are photosynthetic bacteria performing oxygenic photosynthesis (1) and several species can fix atmospheric nitrogen (2). Cyanobacteria are extensively studied for their possible contribution to sustainable production processes (3). They might play a part in a future bioeconomy as solar cell factories due to their ability to grow without organic carbon and nitrogen compounds, even on non-arable land.

Despite these promising features, their effective biotech application is hampered due to low biomass, insufficient product titers, and reaction instabilities (4). However, growing cyanobacteria as biofilms has been demonstrated to be an effective method to address the problem of reaching high cell densities resulting in up to 52 g_cdw_L^−1^ (cdw: cell dry weight) (5), meanwhile systems working with suspended cultures could reach up to 4–8 g_cdw_L^−1^ (4, 6). Biofilms consist of microorganisms embedded in a three-dimensional self-produced extracellular polymeric matrix (EPS). The bacterial communities embedded within the EPS are more resistant to stress conditions like toxic substrates/products. They are self-regenerating which makes them very appealing for biotech applications. Cells in biofilm growing communities are in close contact with each other and have established various inter-species communication strategies, which may affect the community structure (7, 8). Excellent examples of this interaction dynamics are microbial mats, which can be regarded as huge biofilms composed of various specialized microbial layers, including cyanobacteria as primary producers (9).

Recently, capillary biofilm reactors (CBR) have been introduced as an easy-to-maintain system for the study of cyanobacterial biofilms in a biotechnology context (5, 10). The CBR system is a minimized tubular reactor system, which is continuously flushed with medium and thereby represents a plug flow reactor with biomass retention. It can be operated in single or multi-phase mode, the latter involving the introduction of gas segments into the medium flow. These segments serve multiple purpose. They add hydrodynamic forces to the system, which facilitate mass transfer in the biofilm and lead to stronger attachment of the cells to the surface. Moreover, the gas segments extract excess oxygen and, depending on the respective experiment, supply gaseous substrates such as N_2_ or CO_2_ (11).

In previous studies using a CBR, it was demonstrated that various cyanobacteria grow more biomass in the system when co-cultivated with *Pseudomonas taiwanensis* VLB120 (5, 10). Remarkably, *P. taiwanensis VLB120* was using metabolites supplied by the cyanobacterial partner as sole sources of carbon and nitrogen. Among the investigated strains, the filamentous N_2_-fixing cyanobacterium *Tolypothrix* sp. PCC 7712 showed an exceptional performance in terms of biomass formation and cell retention (10).

Here, we report on the spatial organization in an early-stage (young) biofilm composed of *Tolypothrix* sp. PCC 7712 and *P. taiwanensis* VLB120 cultivated in a continuous flow-through system and how different growth conditions influence this spatial organization. Previously established custom-made flow-cells (12) resembling CBR geometry served as confocal scanning laser microscopy (CLSM) compatible cultivation device. CLSM and helium-ion microscopy (HIM) allowed for monitoring the intra-biofilm organization under N_2_-fixing and non-fixing conditions. Based on the microscopic data it was possible to calculate the biovolume of the different species and conclude on population dynamics. The biovolume can be defined as the total volume of all cells, including *Tolypothrix* sp. and *Ps_egfp* cells. We show that *P. taiwanensis* VLB120 is highly important for the initial biofilm attachment, forming a seeding layer directly on the attachment surface. This effect is even more prominent under N_2_-fixing conditions, when the medium is lacking nitrate as a nitrogen source in addition to the organic carbon. Localization of *P. taiwanensis* VLB120 in close proximity to *Tolypothrix* sp. enhanced attachment of the organisms and prevented flush-out upon segmented flow conditions. Shotgun proteomics revealed several *P. taiwanensis* VLB 120 proteins exclusively abundant in N_2_-fixing conditions, which might pose engineering targets to tune biofilm attachment forces in the future.

## MATERIALS AND METHODS

### Chemicals and media

Chemicals were purchased from Sigma-Aldrich (Steinheim, Germany), Merck (Darmstadt, Germany), or Carl-Roth GmbH (Karlsruhe, Germany). *Tolypothrix* sp. PCC 7712 was grown in standard cyanobacteria minimal medium BG11 or BG11-0 (lacking nitrate) (Table S1), while lysogeny broth (LB) complex media and M9 minimal media (13) containing 0.5% (wt/vol) glucose as only energy source were utilized to cultivate *Pseudomonas taiwanensis* VLB120 (Table S2). The Supplementary Doc File 1 describes preparation instructions of the respective media.

### Cultivation and maintenance of microorganisms

*Pseudomonas taiwanensis* VLB120_*egfp* (hereafter referred to as *Ps_egfp*; inhouse strain collection (5), carries a chromosomal integrated constitutive *egfp* gene for synthesis of “enhanced green fluorescence protein” (eGFP) (14) important for fluorescence imaging (see below). *Ps_egfp* was cultivated on LB agar media, prior to overnight cultivation in LB broth (30°C/200 rpm in a Multitron Pro Incubator, Infors HT, Switzerland). Subsequently, 200 µL of pre-culture was transferred to 20 mL M9 minimal medium and cultivated under the same conditions. This cultivation step was repeated once more before mixing the *Ps_egfp* with the cyanobacteria in BG11 or BG11-0, depending on the experiment. Optical density was measured at 450 nm using a spectrophotometer (Libra S11 Visible spectrophotometer, Biochrom, UK) based on previous studies (5, 15, 16).

*Tolypothrix* sp. PCC 7712 (hereafter referred to as *Tolypothrix* sp.) was purchased from Pasteur Culture Collection (PCC). After cultivating it from the -80 cryo stocks on BG11-0 and BG11 agar media with 25 µEm^−2^s^−1^ illumination, it was transferred to BG11-0 or BG11 broth media, depending on the experiment, and cultivated for 5 days under standard conditions (continuous 50 µEm^−2^s^−1^ illumination; 75% humidity) without shaking (Multitron Pro Incubator; Infors HT, Switzerland). Five milliliters of this pre-culture was then transferred to 45 mL fresh BG11-0 or BG11 media, respectively. After 3 days of cultivation under standard conditions, the entire 50 mL *Tolypothrix* sp. culture was suspended by flushing it through 10 mL syringes equipped with stainless steel needles to prevent floc formation. Then, the culture was left in the same media for two more days under standard conditions before mixing with *Ps_egfp*.

### Preparation of the inoculum for flow-cell and CBR

Flow cell and CBR were operated in the same system. Only the cultivation chamber was adjusted (flow cell channel dimensions, 65 mm: 3 mm: 4.5 mm–length: height: width; or capillary dimensions, 200 mm: 3 mm–length: diameter, Fig. S1), while the operation procedure stayed the same. Cyanobacteria cultures were pelleted by centrifugation (5,000 g, 10 min, RT) and resuspended in fresh BG11-0 (for N_2_-fixing conditions) or BG11 media (for non-fixing conditions), respectively by adjusting their final Chl*a* concentration to 8 µM. *Ps_egfp* cultures were washed two times with either BG11-0 or BG11 medium after measuring OD_450_ of the initial culture. Cells were concentrated in the same medium. Final cell concentration was adjusted to OD_450_ to 2.0. All photometric measurements were conducted with diluted samples fitting to the interval between 0.05 and 0.5 to prevent extrapolation, following Lambert–Beer’s law. Consecutively, 5 mL of cyanobacteria culture (8 µM Chl*a*) and 5 mL of *Ps_egfp* culture (OD_450_ of 2) were mixed. After mixing, a final ratio of 4:1 ([Chl*a*]: OD_450_) was obtained and cultures were left for static incubation overnight at standard conditions before inoculating the flow-cells via a syringe through the inoculation port (12). Flow-cells or capillaries were covered with aluminum foil and incubated overnight in the dark. Subsequently, the media flow was started at 52 µL min^−1^ and the system was put under constant illumination at 50–60 µE m^−2^ s^−1^. After 4 days of single-phase flow (only medium), the airflow was started at 52 µL min^−1^ (equal volume as liquid phase).

### Operation of biofilm flow-cells and CBR

Biofilms were cultivated in custom-made flow cells (channel dimensions 65 mm: 3 mm: 4.5 mm–length: height: width) to analyze them via confocal laser scanning microscopy (CLSM) as described previously by David et al. A peristaltic pump (ISM939D; Ismatec, Germany) was used to pump media and filtered air to the system. Two flow-cells, each containing two channels, were operated in parallel: one with BG11-0 for N_2_-fixing conditions; one with BG11 for non-fixing conditions. Both flow-cells were flushed with the respective media for 2 hours for surface conditioning prior to inoculation. CBR experiments (capillary dimensions 200 mm: 2.8 mm–length: diameter) were conducted as described previously (10) with the same conditions applied in flow-cells experiments except for the cultivation period which was 1 month.

### Confocal laser scanning microscopy

Fluorescence imaging was conducted making use of the autofluorescence of Chl*a* and phycocyanin in *Tolypothrix* sp. and the eGFP reporter in *Ps_egfp*. The biofilms were visualized on a confocal laser scanning microscopy (CLSM) (Zeiss LSM 710 NLO, Germany) equipped with laser lines 488 nm (eGFP) and 633 nm (PC + Chl*a*) lasers, and a LD C-Apochromat 10 ×/1.1 W objective. Filters targeting green emission (513–611 nm) and red emission (647–725 nm) was set for the detection. The images were analyzed using IMARIS 8.2 (Bitplane AG, Switzerland).

Each image was divided into three segments in z-dimension: 0–10 µm, 10–20 µm, and 20–30 µm. First, an isosurface was created using the red channel (633 nm excitation) with a quality threshold of over 30. All voxels belonging to the green channel (488 nm excitation) inside the isosurface of the red channel were set to zero via masking feature of the IMARIS 8.2 in order to prevent overestimation of total volume of eGFP cells. Subsequently, masked green channel was used to create another isosurface with a quality threshold of over 10, 12, and 14 for the depth volumes (20–30 µm), (10–20 µm), and (0–10 µm), respectively. Results of the isosurfaces for the different segments of each image were exported as a CVS file, including each isosurface area and their sum values. The derived isosurface based volume data for each image were imported to Origin 2019 (OriginLab Corporation, USA).

Microsoft Excel 2019 (Microsoft, USA) was used to organize the raw data obtained from IMARIS 8.2 before transfer to Origin 2019. Origin 2019 was used for all Whiskers box plots and the calculation of statistical parameters. T-test was applied for the comparison of biovolumes in different depths. Images were taken from six randomly chosen locations from each flow-cells containing two channels.

### Biofilm analysis using helium-ion microscopy

HIM was used to analyze the biofilm grown in the CBR. Silicone was used as capillary material to allow sample preparation for the HIM. Chemical fixation of the biofilms in the capillaries was achieved by pumping 2% paraformaldehyde (PFA) dissolved in cacodylate buffer through the system after 4 days of biofilm cultivation. After the capillaries were filled with the fixation solution, they were clamped tightly and incubated at 4°C overnight. Later, capillaries were gently dehydrated in ethanol series by pumping solutions (10%, 20%, 40%, 60%, 80%, 100%) ethanol in cacodylate buffer into the capillaries and incubating each for 15 min. Then, the ethanol was replaced by a 1:1 mixture of ethanol: hexamethyldisilazane (HMDS) and subsequently pure HMDS, by incubating for 15 min, each followed by air-drying overnight. The dried capillary-samples were cut along the cylinder-axis by vibratome in order to allow for accessing the biofilm on their inside. Finally, the cut-open capillaries were glued onto standard electron microscopy stubs (10 mm) with conductive silver epoxy. Then the samples were ready for HIM imaging. For that a Zeiss Orion NanoFab (Zeiss Peabody, MA, USA) was used. The ion landing energy was set to 25 kV and the beam current amounted to approximately 0.5 pA. For image acquisition secondary electrons were detected with an Everhard–Thornley type electron detector.

### Proteomics sample preparation and data analysis

For proteome analysis, biofilm biomass was removed manually from the CBR by scrubbing the walls with a syringe, and suspending it in 20 mL of BG11-0 or BG11 media, respectively. Four milliliters biofilm suspension was centrifuged (5,000 g/10 min/RT) and the resulting pellet was immediately frozen in liquid nitrogen and kept at −80°C until further processing steps.

Fifty microliters of ammonium bicarbonate solution (50 mM) was added to the frozen sample before it was subjected to three rounds of freeze-thaw followed by 10 min sonication. Centrifugation (10,000 g /5 min/4°C) removed cell debris and the protein containing supernatant was mixed with 50 mM dithiothreitol and incubated in a thermomixer (30°C/1 h/400 rpm). Reduced samples were alkylated by adding 400 mM 2-iodoacetamide (RT/1 h/400 rpm/dark) followed by the addition of 6.3 µg of trypsin (Proteomic Sequencing Grade, Promega GmbH, Germany) and overnight (37°C) incubation. The reaction was halted by adding 1 µL of formic acid (100%). Subsequently, the sample was desalted on a ZipTip-µC_18_ column (Merck Millipore, Merck KGaA, Germany). Eluted samples were dried via vacuum centrifuge.

Extracted and cleaned samples were resuspended in 0.1% formic acid. Analysis was done via nano-liquid chromatography coupled to tandem mass spectrometry (nLC-MS/MS) using an Orbitrap Fusion Trihybrid MS (Thermo Scientific, Rochester, NY, USA) connected to a nano-liquid chromatograph (Dionex Ultimate 3,000RSLC, Thermo Scientific, USA). LC-MS/MS parameters were set according to Seidel et al. (17).

Proteome Discoverer (version 2.2, Thermo Fisher Scientific) was used to identify peptide spectrum matches, peptides and proteins from mass spectrometric analysis. As a search database we used the concatenated NCBI databases for *Pseudomonas* VLB120 (NCBI: txid69328, accession numbers CP003961.1 [genome] and CP003962.1 [plasmid]) and SequestHT as a search engine. Up to two misses of trypsin cleavage were permitted. Mass tolerances of 10.0 ppm and 0.2 Da were accepted for the detection of precursor and fragment ions, respectively. By comparing q values of hits to target and decoy databases, the Target Decoy PSM Evaluator node of Proteome Discoverer evaluated peptide spectrum matches at a false discovery rate (FDR) of 1%. A dynamic modification was chosen as oxidation of methionine residues, and a fixed modification was selected as carbamidomethylation of cysteine residues. Proteome Discoverer’s Minora node was operated to compute protein and peptide abundance values applying intensity-based label-free quantification and utilizing the internal standard Glyceraldehyde-3-phosphate dehydrogenase (GAPDH) obtained from *Staphylococcus aureus*. In Microsoft Excel, protein abundance rankings were generated based on abundance values retrieved from the Proteome Discoverer Minora node. Each protein was ranked within all proteins of a particular sample, with the most abundant protein receiving rank number 1.

## RESULTS

**Fig 1:**
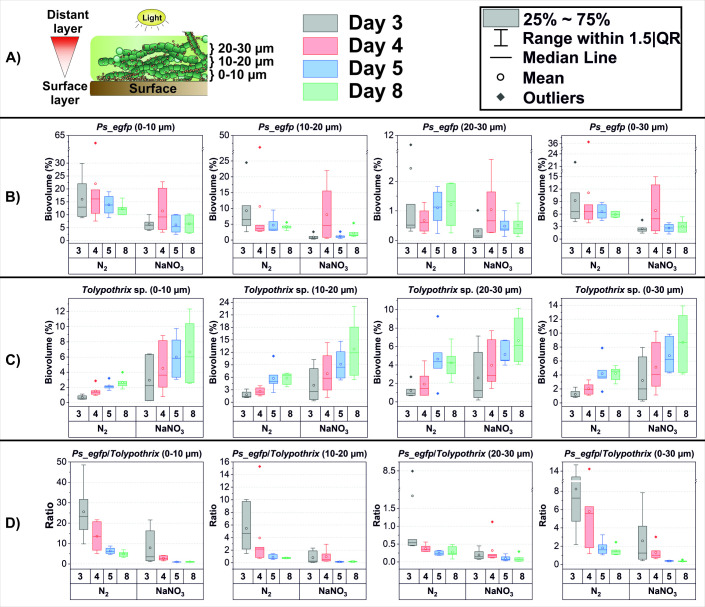
Population dynamics in two species biofilms assessed via CLSM/IMARIS. (**A**) Illustration of FIG. symbols, descriptions, and schematic representation of volume segmentation of the biofilm. Daily biovolume (% cell isosurface/total segment volume) development of (**B**) *Ps_egfp* and (**C**) *Tolypothrix* sp. in different depths and (**D**) their ratios are shown separately as box plots for nitrogen fixing and non-fixing conditions (no of replicates = 6). Total analyzed volume was considered as 100% for each segment. After the image was taken on day 4, segmented flow was started. Day 5 = 24 hours after segmented flow was initiated. Please note the different ranges of the y-axis.

To unravel the population dynamics of dual-species cyanobacterial biofilms during initial attachment, the respective organisms were cultivated in custom made flow-cells and analyzed via CLSM. Biofilm development by *Tolypothrix* sp. PCC 7712 and *Ps_egfp* was mapped via CLSM during the first 8 days of cultivation as dual-species biofilms growing in N_2_-fixing and non-fixing (nitrate containing medium) conditions. Creation of isosurfaces followed by 3-D imaging of the samples allowed for calculation of the biovolumes of *Ps_egfp* and *Tolypothrix* sp. at different depths (10, 20, 30 µm, Fig. 1A) and thus revealed the distribution of the two species over the first 8 days of biofilm development (Fig. 1). Biovolume refers to the total volume of all cells, either *Tolypothrix* sp. or *Ps_egfp*, respectively. *Ps_egfp* was limited in terms of carbon and nitrogen source in every condition. It was found that *Ps_egfp* did not grow in M9 medium when the nitrogen source was changed from NH_4_Cl to NaNO_3_ under aerobic conditions (data not shown). Several *Pseudomonas* species have previously been reported to be capable of the assimilation of nitrate under anaerobic conditions (18 - 20). As demonstrated in a previous study using capillaries containing *Ps_egfp* and *Synechocystis* sp. PCC 6803, anoxic conditions cannot be achieved if organic carbon sources are insufficient for *Ps_egfp* to consume oxygen (5). In previous experiments, *Tolypothrix* sp. PCC 7712 and *Ps_egfp* containing biofilms always accumulated some oxygen in the gas phase (Table S4). The lack of organic carbon source in our experiments means that *Ps_egfp* cannot create an anaerobic environment in which nitrate could be assimilated.

### *Pseudomonas* sp. forms a seeding layer directly on the cultivation surface (0–10 µm)

Especially in the initial phase of biofilm formation *Ps_egfp* was highly abundant in the area close to the attachment surface (0–10 µm). The population did not grow and was maintained at more or less the same level also after segmented flow was started. This is not surprising, as no organic carbon source was available for *Ps_egfp* and organisms were solely relying on the compounds excreted by *Tolypothrix*, which at this time point was not present in high numbers. The surface coverage at 0–10 µm by *Ps_egfp* was in the range of 3–10% after 8 days when cultivated in media containing nitrate as nitrogen source for the cyanobacteria partner (*Ps_egfp* is not capable of using nitrate as nitrogen source under aerobic conditions). Correlating to the distance from the flow cell surface (Fig. 1A), this value decreased to 0.1–1.3% in the most distant layer. Under N_2_-fixing conditions, this observation was even more prominent. *Ps_egfp* maintained a higher total biovolume even after segmented flow started (Day 5), suggesting a stronger attachment compared to nitrate-fed environments (Fig. 1B) which showed a statistically significant difference between (0–10 µm) and (20–30 µm) (Day 5, *P* < 0.05, Table S5). Furthermore, mean values of biovolumes (0–10 µm) were higher by approximately 2-fold in N_2_-fixing conditions after 5 days of cultivation (*P* < 0.05), a trend also visible in the more distant layers. These results clearly demonstrate that the abundance of *Ps_egfp* was reaching up to 100 times higher values close to the attachment surface (0–10 µm) than in deeper layers of the biofilm (20, 30 µm), independent of the applied growth condition.

*Tolypothrix* sp. on the other hand grew linearly in biovolume over the cultivation time of 8 days in all levels analyzed (Fig. 1C). It showed a reverse behavior compared to *Ps_egfp*, with increasing abundance towards the outer levels of the biofilm closer to the light source. When nitrate was added to the medium, deviations in biofilm biovolume values was enhanced and values fluctuated significantly (Fig. 1C) which resulted in statistically non-significant comparisons (*P* > 0.05, Table S5). Nevertheless, also under these conditions a linear increase in mean values of *Tolypothrix* sp. biovolume could be detected. The results show that *Tolypothrix* sp. grows faster in nitrate-fed biofilms, but is more stable when fixing molecular nitrogen. The uptake of nitrate is a less costly process for cyanobacteria than N_2_-fixation. Therefore, it was expected that cyanobacteria would grow faster in nitrate-fed biofilms. However, the big deviation observed in the biovolume content in the nitrate-fed biofilms indicate that there are strong fluctuations in detachment events under these conditions.

The ratios of *Ps_egfp : Tolypothrix sp*. (*Ps : To*) reflect the above described findings (Fig. 1D). Surprisingly, *Ps_egfp* outcompetes *Tolypothrix* in terms of biovolume during this initial biofilm development stage, especially directly at the surface layer, where the ratio *Ps : To* is still 5:1 under N_2_-fixing conditions after 8 days of cultivation (*P* < 0.05, Table S5). However, for all conditions and in all levels analyzed, the initially high ratio of *Ps : To* is decreasing over time, especially after segmented flow was started and it is to be expected, that in the course of biofilm development *Tolypothrix* will be the prominent organism.

### Co-localized cells resisting to the segmented flow

Segmented flow is an important feature of operating CBRs and has been shown to be beneficial for biofilm development in previous studies (5, 10, 11). Therefore, the impact of segmented flow on the spatial localization of the organisms relative to each was analyzed.

Starting the airflow on day 4 and operating the system under segmented flow conditions promoted biofilm stability and reduced biovolume fluctuations significantly. Under N_2_-fixing conditions, the air segments not only increased hydrodynamic forces but also delivered molecular nitrogen for the nitrogen fixing reaction (Table S4), resulting in a more stable biofilm and an increase in *Tolypothrix* sp. biovolume (10). As a result of segmented flow, mean values of *Ps_egfp* biovolume of decreased and remained stable for three more days (Days 5–8) in nitrate-fed biofilms. This effect was especially obvious in the lower levels of the biofilm where *Ps_egfp* was most abundant. However, N_2_-fixing biofilms demonstrated greater resilience to segmented flow by maintaining biovolumes more or less at a constant level before and after segmented flow started.

**Fig 2:**
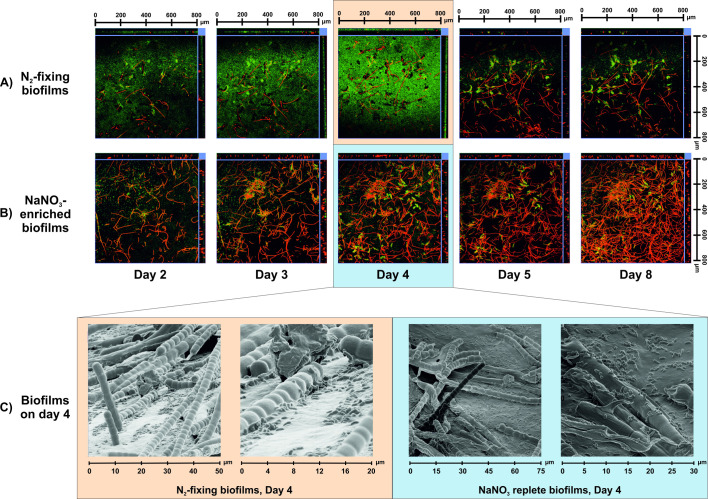
Spatial localization in an early stage biofilm of *Tolypothrix* sp. and *Ps_egfp*. After imaging on day 4, segmented flow was started. (**A**) Shows CLSM images taken from biofilms cultivated in N_2_-fixing conditions and (**B**) in media supplemented with nitrate as nitrogen source; both cultivated in a flow-cell. (**C**) HIM images of 4-day-old mixed species biofilms grown in CBRs. Blue square/lines in each image correspond to respective cross-sections.

After 3–4 days of cultivation, *Ps_egfp* cells started to surround the filaments of *Tolypothrix* sp. (Fig. 2; Fig. S2). This co-localization behavior was more prominent in N_2_-fixing conditions (Fig. 2A), yet it was also detectable in nitrate-fed biofilms (Fig. 2B). For better resolution, this experiment was repeated in the CBR and the biofilm was analyzed via HIM. In the HIM micrographs, *Ps_egfp* cells attached to the EPS structure around *Tolypothrix* sp. filaments are visible (Fig. 2C) on day 4 of biofilm cultivation. Moreover, a layer of *Ps_egfp* embedded in the EPS matrix covers the attachment surface, supporting the conclusion on the conditioning film formation of *Ps_egfp*.

**Fig 3:**
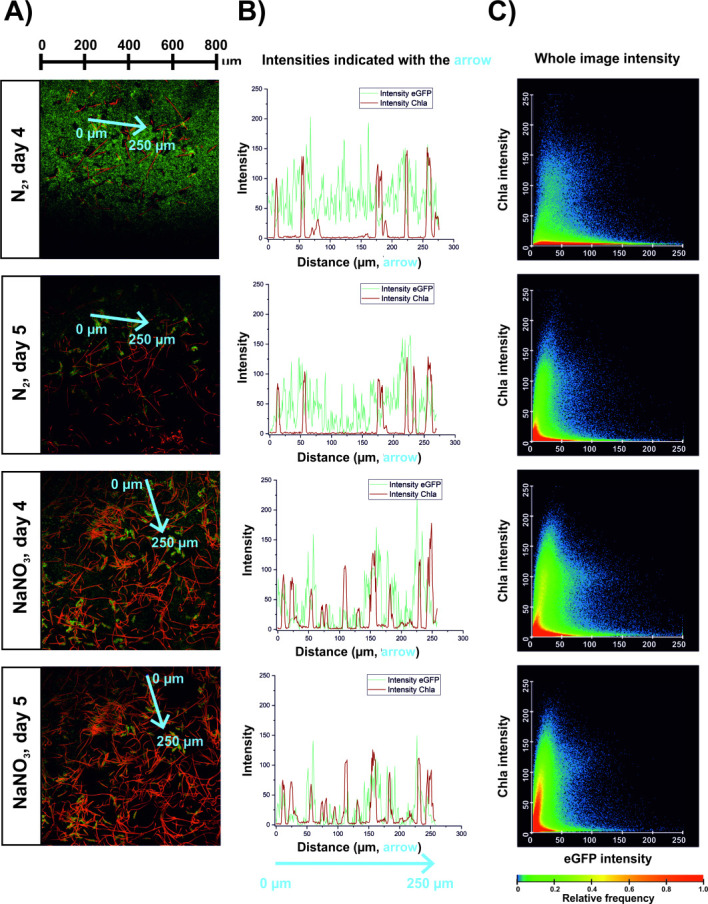
Influence of segmented flow on early stage biofilm of *Tolypothrix* sp. and *Ps_egfp* analyzed via spatially resolved fluorescence intensity patterns. (**A**) CLSM images are shown on the left side and white arrows indicating co-localization areas to be visualized in (**B**) as an intensity graph. (**C**) Whole image fluorescence intensity.

Surprisingly, after starting the segmented flow on day 4, *Ps_egfp* localized in immediate vicinity of *Tolypothrix* sp. stayed fixed in their position, while other *Ps_egfp* were flushed out in significant numbers (Fig. 2A and B; Day 5; Fig. 3A). Before segmented flow started, green fluorescence was observed randomly in almost every position indicated by the blue arrow in Fig. 3, on the contrary, red fluorescence emitted by *Tolypothrix* sp. showed intensities only at certain positions (Fig. 3B). After the segmented flow started, the high red fluorescence intensities did not change the position pattern in contrast to the green fluorescent signal. These drastically reduced especially in the positions where no co-localization with red fluorescing *Tolypothrix* sp. occurred. Although this behavior was more pronounced when *Tolypothrix* sp. had to fix molecular nitrogen, it was also observed in the presence of NaNO_3_. *Ps_egfp* cells were apparently stronger attached when being localized in close proximity of *Tolypothrix* sp. Thus, segmented flow stabilized the overall biofilm and reduced the variations in biovolume values by flushing out loosely attached cells from the system. Furthermore, whole-image intensities showed *Tolypothrix* sp. fluorescence intensities increasing after the start of segmented flow indicating boosted cyanobacterial growth (Fig. 3C).

### Surface and substrate binding proteins found in N_2_-fixing biofilms

Shotgun proteomics were conducted to further access the effect N_2_-fixation has on cell attachment. To gain enough biovolume, the CBR system was used for cultivation and biofilms were cultivated for 1 month before harvest. As both species of the consortium have been completely sequenced ((21) ; Genbank accession: GCA_025860405.1 and GCA_000494915.1 ), it was possible to allocate many of the proteins to the respective species. Here, we focused on proteins originating from *Ps_egfp* to find possible explanations regarding microscopy results. In this context, several proteins originating from *Ps_egfp* were identified only in N_2_-fixing biofilms but not in nitrate-amended biofilms (Table 1), including some related to cell-cell or cell-surface interaction.

**TABLE 1:** Proteins of *Ps_egfp* abundant in N_2_-fixing dual-species biofilms media (FDR of peptide sequences was adjusted to <1%)^*a*^

locus_tag	Proteins	Average rankings		PSM				Protein FDR confidence			
		M w/o N	M w/ N	M1 w/o N	M2 w/o N	M1 w/ N	M2 w/ N	M1 w/o N	M2 w/o N	M1 w/ N	M2 w/ N
PVLB_23220	PrkA family serine protein kinase	1,653 ± 21.2	ND	1	3	1		H	H	H	ND
PVLB_16155	Potassium-transporting AT Pase subunit B	222.5 ± 29	ND	2	1			H	H	ND	ND
PVLB_21235	OmpA family outer membrane protein	1,568.5 ± 37.5	ND	2	1			H	H	ND	ND
PVLB_04415	Ubiquinol-cytochrome c reductase, cytochrome c1	1,361.5 ± 48.8	ND	2	2			H	H	ND	ND
PVLB_01645	Malate dehydrogenase	1,468.5 ± 81.3	ND	2	3		1	H	H	ND	H
PVLB_02140	Poly(hydroxyalkanoate) granule-associated protein	1,487 ± 101.8	ND	3	1			H	H	ND	ND
PVLB_01155	LytTR family two component transcriptional regulator	1,732.5 ± 102.5	ND	1	1		1	H	H	ND	H
PVLB_02145	PhaF protein	673.5 ± 159.1	ND	4	6			H	H	ND	ND
PVLB_23550	Pyruvate dehydrogenase subunit E1	969 ± 162.6	ND	5	6		1	H	H	ND	M
PVLB_23795	Glycine betaine/L-proline ABC transporter periplasmic binding protein	847 ± 251.7	ND	4	8			H	H	ND	ND
PVLB_08345	Transcriptional regulator CysB	1,386.5 ± 344.4	ND	3	3			H	H	ND	ND
PVLB_21320	Lipoprotein	670.5 ± 372.6	ND	1	4			H	H	ND	ND
PVLB_05585	OmpA/MotB domain-containing protein	1,668 ± 400.2	ND	1	2			H	H	ND	ND
PVLB_06300	Extracellular solute-binding protein	874.5 ± 425	ND	2	11		1	H	H	ND	H
PVLB_02735	HflK protein	1,158.5 ± 221.3	ND	4	6		1	H	H	ND	H
PVLB_05670	Leucine ABC transporter subunit substrate-binding protein LivK	394 ± 60.8	948 ± 116	11	14	6	6	H	H	H	H
PVLB_05350	Extracellular solute-binding protein	798.5 ± 195.9	ND	3	6	1	1	H	H	H	H
PVLB_15240	Transcriptional regulator MvaT, P16 subunit	1,063 ± 60.8	ND	3	3	1		H	H	H	ND
PVLB_01935	Glutamine synthetase	422 ± 93.3	1,100.5 ± 71.4	8	10	3	3	H	H	H	H
PVLB_20160	Arginine deiminase	629.5 ± 33.2	1,359 ± 121.6	9	11	3	3	H	H	H	H
PVLB_25325	Pyruvate carboxylase subunit B	373 ± 8.5	1,177.5 ± 57.3	14	11	2	2	H	H	H	H
PVLB_24595	Putrescine ABC transporter periplasmic putrescine- binding protein	468.5 ± 88.4	1,283.5 ± 58.7	9	9	2	3	H	H	H	H
PVLB_24275	LysM domain/BON superfamily protein	1,039 ± 83.4	ND	2	3		2	H	H	ND	H
PVLB_23145	Extracellular solute- binding protein	651.5 ± 62.9	ND	7	7	1	1	H	H	H	H
PVLB_02885	Extracellular ligand- binding receptor	765 ± 314	1,854 ± 178.2	3	6	2	1	H	H	H	H
PVLB_25330	Pyruvate carboxylase subunit A	547 ± 90.5	1,643.5 ± 253.9	9	10	1	3	H	H	H	H
PVLB_16295	2-oxoglutaratedehydrogenase E1 component	915 ± 42.4	ND	6	3		2	H	H	ND	H

^*a*^ Full list is given in Supplementary Data Set 1. M1 and M2 represent biological replicates, and nitrate-fed and lacking biofilms are indicated with “w N” and “w/o N,” respectively. Total number of proteins were 2,315 (M1 w/o N), 2,324 (M2 w/o N), 2,256 (M1 w N), and 2,294 (M2 w N). H, high; ND, not determined; PSM, peptide-spectrum match.

The OmpA family protein was identified in previous studies to be important for outer membrane integrity and resistance against environmental stress (22). The peptidoglycan binding LysM domain contains a motif which binds to cell walls and it is commonly found as “cell wall binding domain” in several species (23). LysM domains can recognize and bind several different ligands containing N-acetylglucosamine (24)().

Moreover, the extracellular binding protein (PVL_06300) was detected exclusively in N_2_-fixing conditions yet similar proteins were identified in NaNO_3_ fed biofilms as well. Other interesting proteins detected in only N_2_-fixing biofilms include malate dehydrogenase (PVLB_01645), PhaF protein (PVLB_02145), and polyhydroxyalkanoate (PHA) granule-associated protein (PVLB_02140). Possible reasons behind their abundances in this specific condition are discussed in detail in the next section.

Furthermore, the detachment of *Ps_egfp* was monitored in the CBR via plating (track-dilution method [24], Table S3) of the flow through followed by colony counts. Around 2.92 × 10^8^ ± 3.20 × 10^7^ and 2.03 × 10^9^ ± 2.09 × 10^8^ CFU*_Ps_egfp_*/L have been detected for N_2_-fixing and nitrate-amended conditions, respectively, after 1 mo of cultivation. *Ps_egfp* colonies were quantified also in the biofilm, resulting in 1.42 × 10^11^ ± 1.36 × 10^10^ and 3.48 × 10^11^ ± 1.48 × 10^10^ CFU*_Ps_egfp_* /L, respectively. In spite of the fact that nitrate-fed conditions produced more *Ps_egfp* cells in the biofilm after 1 month of cultivation, the ratio of resident to detached cells in N_2_-fixing conditions was almost twice as high than that in nitrate-fed conditions (Table S3). This supports the findings of the proteomics and microscopy studies that attachment of *Ps_egfp* is stronger in conditions involving N_2_-fixing by *Tolypothrix* sp. cells.

## DISCUSSION

The main driver of this work was the observation, that in an artificial environment like a CBR, cyanobacteria develop much better biofilms in terms of biomass when co-cultivated with the chemo-heterotrophic organism *Pseudomonas taiwanensis* VLB120 (5, 10). The respiration activity of *Ps_egfp* leading to low oxygen tension in the system, was identified as a main reason for this phenomenon (5), albeit not the only one. Exchanging *Ps_egfp* with other aerobic heterotrophs like *E. coli* did not lead to enhanced biofilm formation (10). *P. taiwanensis* VLB 120 is a well described biofilm-forming organism applied in various biotechnological applications (11, 25, 26). The identification of possible communication mechanisms between two species was so far not successful (data not shown). Based on the previously published studies we hypothesized that *P. taiwanensis* forms a kind of seeding carpet on the first layer of the attachment surface and thereby facilitates cell attachment and biofilm growth of the cyanobacterial partner. The in-depth imaging and analysis of the initial biofilm formation phase presented here supported this hypothesis. The level directly above the attachment surface (0, 10 µm) was dominated by *Ps_egfp* cells, independent of whether *Tolypothrix* sp. was depending on nitrogen fixation or not. The *Ps_egfp* biovolume was almost 100 times larger directly on the attachment surface as compared to deeper levels (20, 30 µm). The results of this study clearly indicate that *Ps_egfp* promotes general surface attachment of consortia in the early growth phase, despite the fact that this organism is heavily limited in carbon and nitrogen compounds, respectively.

Under N_2_-fixing conditions, the overall biovolume development was less fluctuating indicating strong attachment especially of the cyanobacterial partner, although nitrate-fed biofilms had higher total biovolume values. These findings are in line with a previously published study, where it was shown, that also other cyanobacteria like *Nostoc* sp. had lower detachment rates under N_2_-fixing conditions (10). In a complementary CBR experiment cultivated for 1 month, it was observed that ratio of resident to detached *Ps_egfp* cells was elevated 2-fold in N_2_-fixing biofilms compared to nitrate-fed biofilms (Table S3). These findings are very interesting in terms of keeping the harvested energy in the cultivated system. Further studies should focus on modelling of these systems in order to understand the amount of energy being lost comparing N_2_-fixing and non-fixing conditions. It may be speculated that the specialized surface layers of the heterocysts, the specialized cells where the N_2_-fixing enzymes are located in many cyanobacterial species, are important for the enhanced attachment. As nitrogenase is highly O_2_-sensitive, heterocysts have special surface layers, which provides an O_2_-free inner compartment. According to some studies, these surface layers contain a variety of biopolymers that are responsible for protecting the structure (27, 28). It may be that these biopolymers facilitate cell attachment not only of the cyanobacterium, but also in case of the *Ps_egfp*. Fitting to this hypothesis is the fact, that also *Ps_egfp* seems to attach much stronger to the surface in close proximity of *Tolypothrix* sp. cells.

Surprisingly, *Ps_egfp* outcompeted *Tolypothrix* in total biovolumes in the early stage biofilms in all conditions and in all levels analyzed, especially under N_2_-fixing conditions. At a first glance, this may be surprising, as *Ps_egfp* is limited not only in the carbon, but also in the nitrogen source. The synthesis of EPS seems to be enhanced when nitrogen is depleted in some cases like *Anabaena* sp. ATCC 33047 (29) and *Cyanothece* sp (30). It is likely that this is related to the rise in the C:N ratio, which facilitates the addition of carbon to polymers (31 - 33), which in turn might lead to an increase in *Ps_egfp* cells.

In the context of cell attachment, the hydrodynamic forces introduced into the CBR by the addition of the air segments are highly important (34). Following the start of segmented flow at day 4 of cultivation, biofilms became generally more stable in terms of showing fewer fluctuations and biovolume development was strongly enhanced under all cultivation conditions tested. The hydrodynamic force introduced into the system by the air segments might have resulted in a change in overall biofilm structure by washing out loosely attached cells (11), and an increase in biovolume of *Tolypothrix* sp. (Fig. 2 and 3). Under N_2_-fixing conditions, additional molecular nitrogen was introduced into the system via the air bubbles, promoting the growth of *Tolypothrix* sp. cells. Furthermore, the co-localized clusters of *Ps_egfp* cells surrounding *Tolypothrix* sp. filaments showed a strong attachment after segmented flow was initiated. These results are in agreement with other publications, showing that segmented flow increases the surface coverage and cell attachment in CBRs (5, 10, 11).

Proteomics revealed the abundance of a wide variety of proteins in *Ps_egfp*, which were only abundant under N_2_-fixing conditions. Of particular interest are the LysM-domain protein, a protein belonging to the OmpA family, a malate dehydrogenase, and some proteins related to PHA synthesis. The LysM-domain protein recognizes and binds several different peptidoglycan ligands that contain N-acetylglucosamine (35), identified in ,for example, *Nostoc punctiforme* and *Anabaena cylindrica* fixing nitrogen (36 - 38). A comparable trait in *Tolypothrix* sp. might explain the strong co-localization of *Ps_egfp* and *Tolypothrix* sp. in N_2_-fixing conditions. Furthermore, plants can identify their symbiotic microbes via specific LysM domains (35). It is possible that *Ps_egfp* and *Tolypothrix* sp. have a similar interaction that could explain why in N_2_-fixing biofilms superior attachment and co-localization as compared to nitrate-feeding biofilms was observed. More experimental evidence is required to support this claim.

Also interesting is a protein candidate from the OmpA family, which also was abundant only in N_2_-fixing biofilms. A recent study showed that upon deletion of the peptidoglycan-binding anchor (Pba) of the OmpA family protein *P. aeruginosa* formed low-density, unorganized biofilms which were very fragile and sensitive to shaking compared to the wild type (39). In addition, some studies have demonstrated that the OmpA family of proteins can act as a pore for the diffusion of small molecules (40 - 42). *Ps_egfp* might use this protein in order to increase the diffusion of necessary nutrients and also attach strongly to surfaces in N_2_-fixing biofilms.

Malate dehydrogenase, catalyzing the bilateral conversion of L-malate and oxaloacetate in the tricarboxylic acid cycle (TCA), is another enzyme of *Ps_egfp* that was detected to be abundant in N_2_-fixing biofilms. Its abundance under N_2_-fixing conditions is interesting because its synthesis is triggered by altering conditions like oxygenation and the type of carbon substrates available (43, 44). Abundance of this protein in *Ps_egfp* might indicate that N_2_-fixing cyanobacteria release different carbon substrates which subsequently serve as feedstock for *Ps_egfp* in the biofilm compared to nitrate-assimilating *Tolypothrix* sp. cells.

Additionally, proteomics revealed that proteins related to PHA synthesis were abundant in only N_2_-fixing biofilms such as PhaF (PVLB_02145) and the PHA granule-associated protein (PVLB_02140). In some bacteria, such as *Pseudomonas*, carbon is stored as PHA, which is composed of 6–14 carbon atoms (45). Excess carbon availability but also certain nutrient limitations may promote medium-chain PHA accumulation in *Pseudomonas* strains (46). There are numerous studies reporting on PHA production in *Pseudomonas sp*. is strongly dependent on the type and amount of organic acids and nitrogen starvation (47 - 49). Interestingly, *Tolypothrix* sp. can produce fatty acids like γ-linolenic acid, and palmitic acid (50). Combined with nitrogen starvation this may induce the respective enzymes related to PHA synthesis. However, as *Ps_egfp* is severely limited in our biofilm, no significant PHA synthesis is to be expected. Kelly (51) demonstrated that pyruvate dehydrogenase E1 and peptidoglycan associated lipoprotein interact with PhaF protein and localize to PHA granules *in vivo*. A similar protein profile was also observed in our study under N_2_-fixing conditions in biofilms composed of *Tolypothrix* sp. and *Ps_egfp*.

### Conclusion

This study contributes to a better understanding of microbial interactions in artificial dual-species phototrophic biofilms containing the cyanobacterium *Tolypothrix* sp. PCC 7712 and *P. taiwanensis* VLB 120_*egfp* as supporter strain in a continuous cultivation system. It was possible to show, that *Ps_egfp* established a conditioning film on the attachment surface, despite being strongly C and N limited, facilitated the attachment of cyanobacteria partner. N_2_-fixing biofilms showed higher *Ps_egfp* to *Tolypothrix* sp. ratios during initial attachment period indicating stronger attachment of *Ps_egfp* in this condition. In this context, a CBR experiment showed three times higher ratio of residing to detached cells in N_2_-fixing biofilms compared to nitrate-fed biofilms in the later phase of biofilms. Additionally, co-localization of *Ps_egfp* and *Tolypothrix* sp. in close proximity prevented detachment of *Ps_egfp*, which was correlated with the identification of several proteins of higher abundance being relevant for cell attachment. In conclusion, showing the role of *Ps_egfp s* in the initial attachment process, as well as the effects of different nitrogen feeding strategies and hydrodynamic forces, may assist in optimizing biofilm photobioreactors and facilitate the application of cyanobacteria for productive processes.

## SUPPLEMENTAL MATERIAL

Fig. S1 msystems.00302-23-s0001.tif Illustration of CBR and flow cell system (modified from Bozan et al. (2022)). They both have the same system except the cultivation chamber.

Fig. S2 msystems.00302-23-s0002.tif Additional CLSM images show the co-localization of species in different locations. (A) shows N_2_-fixing biofilms on day 4, (B) indicates NaNO_3_-fed biofilms. Images indicated with the yellow box were taken in a different experiment independent from the current study but they were also same conditions.

Table S1 msystems.00302-23-s0003.docx Media solutions and their ingredients.

Table S2 msystems.00302-23-s0004.docx
*Ps_egfp* media solutions.

Table S3 msystems.00302-23-s0005.docx Colony counts for determining numbers of *Pseudomonas*. Number of *Ps_egfp* colonies in the biofilm and outflow at day 29 together with their ratios. M1 and M2 represent biological replicates of each condition; 'w/o N' represents the N_2_-fixing, and 'w/ N' represents non-fixing biofilms.

Table S4 msystems.00302-23-s0006.docx Gas composition of CBRs containing *Tolypothrix* sp. PCC 7712 and *Ps_egfp* after 13 days of cultivation. Observations were made using CBRs containing *Tolypothrix* sp. PCC 7712 and *Ps_egfp* in nitrate-fed and N_2_-fixing conditions after 13 days of cultivation to demonstrate that there were no anaerobic conditions present throughout biofilm cultivation, where Nitrate could have been assimilated by *Ps_egfp*. Table S4 illustrates the gas measurement conducted via GC (method description described in Bozan et al., 2022).

Table S5 msystems.00302-23-s0007.docx T-test results from the comparison of differences between (a) changing depths and (b) NaNO_3_-fed and N_2_-fixing biofilms in a certain volume.

Data Set S1 msystems.00302-23-s0008.xlsx Complete proteomics data. Full list of *Ps_egfp* and *Tolypothrix* sp. proteins abundant N_2_-fixing and NaNO_3_-fed dual-species biofilms (FDR of peptide sequences was adjusted to < 1%) are shown in the Excel file. M1 and M2 represent biological replicates of each condition; 'w/o N' represents the N_2_-fixing, and 'w/ N' represents non-fixing biofilms.

Medium preparation procedure msystems.00302-23-s0009.docx Preparation procedure for the media used in the study.

ASM does not own the copyrights to Supplemental Material that may be linked to, or accessed through, an article. The authors have granted ASM a non-exclusive, world-wide license to publish the Supplemental Material files. Please contact the corresponding author directly for reuse.

## References

[1] Stanier RY , Cohen-Bazire G . 1977. Phototrophic prokaryotes: the cyanobacteria. Annu Rev Microbiol 31:225–274. doi:10.1146/annurev.mi.31.100177.001301 410354

[2] Kasting JF , Siefert JL . 2002. Life and the evolution of earth’s atmosphere. Science 296:1066–1068. doi:10.1126/science.1071184 12004117

[3] Jodlbauer J , Rohr T , Spadiut O , Mihovilovic MD , Rudroff F . 2021. Biocatalysis in green and blue: cyanobacteria. Trends Biotechnol 39:875–889. doi:10.1016/j.tibtech.2020.12.009 33468423

[4] Posten C . 2009. Design principles of photo-bioreactors for cultivation of microalgae. Eng Life Sci 9:165–177. doi:10.1002/elsc.200900003

[5] Hoschek A , Heuschkel I , Schmid A , Bühler B , Karande R , Bühler K . 2019. Mixed-species biofilms for high-cell-density application of Synechocystis sp. PCC 6803 in capillary reactors for continuous cyclohexane oxidation to cyclohexanol. Bioresour Technol 282:171–178. doi:10.1016/j.biortech.2019.02.093 30861446

[6] Lippi L , Bähr L , Wüstenberg A , Wilde A , Steuer R . 2018. Exploring the potential of high-density cultivation of cyanobacteria for the production of cyanophycin. Algal Res 31:363–366. doi:10.1016/j.algal.2018.02.028

[7] Flemming H-C , Wingender J . 2010. The biofilm matrix. Nat Rev Microbiol 8:623–633. doi:10.1038/nrmicro2415 20676145

[8] Halan B , Buehler K , Schmid A . 2012. Biofilms as living catalysts in continuous chemical syntheses. Trends Biotechnol 30:453–465. doi:10.1016/j.tibtech.2012.05.003 22704028

[9] Bolhuis H , Cretoiu MS , Stal LJ . 2014. Molecular ecology of microbial mats. FEMS Microbiol Ecol 90:335–350. doi:10.1111/1574-6941.12408 25109247

[10] Bozan M , Schmid A , Bühler K . 2022. Evaluation of self-sustaining cyanobacterial biofilms for technical applications. Biofilm 4:100073. doi:10.1016/j.bioflm.2022.100073 35434604 PMC9006728

[11] Karande R , Halan B , Schmid A , Buehler K . 2014. Segmented flow is controlling growth of catalytic biofilms in continuous multiphase microreactors. Biotechnol Bioeng 111:1831–1840. doi:10.1002/bit.25256 24729096

[12] David C , Heuschkel I , Bühler K , Karande R . 2020. Cultivation of productive biofilms in flow reactors and their characterization by CLSM, p 437–452. In Immobilization of enzymes and cells. Humana Press, New York. doi:10.1007/978-1-0716-0215-7 31939142

[13] Sambrook J , Russell DW . 2001. In Molecular cloning: a laboratory manual. Cold 577 Spring Harbor Laboratory, New York.

[14] Zhang G , Gurtu V , Kain SR . 1996. An enhanced green fluorescent protein allows sensitive detection of gene transfer in mammalian cells. Biochem Biophys Res Commun 227:707–711. doi:10.1006/bbrc.1996.1573 8885998

[15] Heuschkel I , Dagini R , Karande R , Bühler K . 2020. The impact of glass material on growth and biocatalytic performance of mixed-species biofilms in capillary reactors for continuous cyclohexanol production. Front Bioeng Biotechnol 8:588729. doi:10.3389/fbioe.2020.588729 33042983 PMC7522790

[16] Bretschneider L , Heuschkel I , Bühler K , Karande R , Bühler B . 2022. Rational orthologous pathway and biochemical process engineering for adipic acid production using Pseudomonas taiwanensis VLB120. Metab Eng 70:206–217. doi:10.1016/j.ymben.2022.01.014 35085781

[17] Seidel K , Kühnert J , Adrian L . 2018. The complexome of Dehalococcoides mccartyi reveals its organohalide respiration-complex is modular. Front Microbiol 9:1130. doi:10.3389/fmicb.2018.01130 29946299 PMC6005880

[18] Samuelsson MO . 1985. Dissimilatory nitrate reduction to nitrate, nitrous oxide, and ammonium by Pseudomonas putrefaciens. Appl Environ Microbiol 50:812–815. doi:10.1128/aem.50.4.812-815.1985 4083881 PMC291753

[19] Almeida JS , Reis MA , Carrondo MJ . 1995. Competition between nitrate and nitrite reduction in denitrification by Pseudomonas fluorescens. Biotechnol Bioeng 46:476–484. doi:10.1002/bit.260460512 18623340

[20] Fewson CA , Nicholas DJD . 1961. Nitrate reductase from Pseudomonas aeruginosa. Biochim Biophys Acta 49:335–349. doi:10.1016/0006-3002(61)90133-0 13699254

[21] Bozan M , Popp D , Kallies R , Rocha UN , Klähn S , Bühler K . 2022. Whole-genome sequence of the filamentous diazotrophic cyanobacterium Tolypothrix sp. PCC 7712 and its comparison with non-diazotrophic Tolypothrix sp. PCC 7601. Front Microbiol 13:1–12. doi:10.3389/fmicb.2022.1042437 PMC9679502 36425037

[22] Wang Y . 2002. The function of OmpA in Escherichia coli. Biochem Biophys Res Commun 292:396–401. doi:10.1006/bbrc.2002.6657 11906175

[23] Visweswaran GRR , Leenhouts K , van Roosmalen M , Kok J , Buist G . 2014. Exploiting the peptidoglycan-binding motif, LysM, for medical and industrial applications. Appl Microbiol Biotechnol 98:4331–4345. doi:10.1007/s00253-014-5633-7 24652063 PMC4004799

[24] Jett BD , Hatter KL , Huycke MM , Gilmore MS . 1997. Simplified agar plate method for quantifying viable bacteria. Biotechniques 23:648–650. doi:10.2144/97234bm22 9343684

[25] Gross R , Hauer B , Otto K , Schmid A . 2007. Microbial biofilms: new catalysts for maximizing productivity of long-term biotransformations. Biotechnol Bioeng 98:1123–1134. doi:10.1002/bit.21547 17614329

[26] Schmutzler K , Schmid A , Buehler K . 2015. A three-step method for analysing bacterial biofilm formation under continuous medium flow. Appl Microbiol Biotechnol 99:6035–6047. doi:10.1007/s00253-015-6628-8 25936379

[27] Zhou R , Wolk CP . 2003. A two-component system mediates developmental regulation of biosynthesis of a heterocyst polysaccharide. J Biol Chem 278:19939–19946. doi:10.1074/jbc.M300577200 12637541

[28] Kumar K , Mella-Herrera RA , Golden JW . 2010. Cyanobacterial heterocysts. Cold Spring Harb Perspect Biol 2:a000315. doi:10.1101/cshperspect.a000315 20452939 PMC2845205

[29] Moreno J , Vargas MA , Olivares H , Rivas J , Guerrero MG . 1998. Exopolysaccharide production by the cyanobacterium Anabaena sp. ATCC 33047 in batch and continuous culture. J Biotechnol 60:175–182. doi:10.1016/S0168-1656(98)00003-0

[30] De Philippis R , Margheri MC , Pelosi E , Ventura S . 1993. Exopolysaccharide production by a unicellular cyanobacterium isolated from a hypersaline habitat. J Appl Phycol 5:387–394. doi:10.1007/BF02182731

[31] Otero A , Vincenzini M . 2003. Extracellular polysaccharide synthesis by Nostoc strains as affected by N source and light intensity. J Biotechnol 102:143–152. doi:10.1016/s0168-1656(03)00022-1 12697392

[32] Kumar AS , Mody K , Jha B . 2007. Bacterial exopolysaccharides – a perception. J Basic Microbiol 47:103–117. doi:10.1002/jobm.200610203 17440912

[33] Pereira S , Zille A , Micheletti E , Moradas-Ferreira P , De Philippis R , Tamagnini P . 2009. Complexity of cyanobacterial exopolysaccharides: composition, structures, inducing factors and putative genes involved in their biosynthesis and assembly. FEMS Microbiol Rev 33:917–941. doi:10.1111/j.1574-6976.2009.00183.x 19453747

[34] Kashid MN , Gerlach I , Goetz S , Franzke J , Acker JF , Platte F , Agar DW , Turek S . 2005. Internal circulation within the liquid slugs of a liquid−liquid slug-flow capillary microreactor. Ind Eng Chem Res 44:5003–5010. doi:10.1021/ie0490536

[35] Gust AA , Willmann R , Desaki Y , Grabherr HM , Nürnberger T . 2012. Plant LysM proteins: modules mediating symbiosis and immunity. Trends Plant Sci 17:495–502. doi:10.1016/j.tplants.2012.04.003 22578284

[36] Tien C-J , Sigee DC , White KN . 2005. Characterization of surface sugars on algal cells with fluorescein isothiocyanate-conjugated lectins. Protoplasma 225:225–233. doi:10.1007/s00709-005-0092-8 16228900

[37] Schüßler A , Meyer T , Gehrig H , Kluge M . 1997. Variations of lectin binding sites in extracellular glycoconjugates during the life cycle of Nostoc punctiforme, a potentially endosymbiotic cyanobacterium. Euro J Phycol 32:233–239. doi:10.1017/S0967026297001340

[38] Qiu Y , Tian S , Gu L , Hildreth M , Zhou R . 2019. Identification of surface polysaccharides in akinetes, heterocysts and vegetative cells of Anabaena cylindrica using fluorescein-labeled lectins. Arch Microbiol 201:17–25. doi:10.1007/s00203-018-1565-4 30173343

[39] Paulsson M , Kragh KN , Su Y-C , Sandblad L , Singh B , Bjarnsholt T , Riesbeck K . 2021. Peptidoglycan-binding anchor is a Pseudomonas aeruginosa OmpA family lipoprotein with importance for outer membrane vesicles, biofilms, and the periplasmic shape. Front Microbiol 12:639582. doi:10.3389/fmicb.2021.639582 33717034 PMC7947798

[40] Sugawara E , Nikaido H . 1992. Pore-forming activity of Ompa protein of Escherichia coli. J Biol Chem 267:2507–2511. doi:10.1016/S0021-9258(18)45908-X 1370823

[41] Negoda A , Negoda E , Reusch RN . 2010. Oligo- (R) -3-hydroxybutyrate modification of sorting signal enables pore formation by Escherichia coli OmpA. Biochim Biophys Acta 1798:1480–1484. doi:10.1016/j.bbamem.2009.11.023 20004640 PMC2890286

[42] Negoda A , Negoda E , Reusch RN . 2010. Resolving the native conformation of Escherichia coli OmpA. FEBS J 277:4427–4437. doi:10.1111/j.1742-4658.2010.07823.x 21069910 PMC3059720

[43] Park SJ , Cotter PA , Gunsalus RP . 1995. Regulation of malate dehydrogenase (mdh) gene expression in Escherichia coli in response to oxygen, carbon, and heme availability. J Bacteriol 177:6652–6656. doi:10.1128/jb.177.22.6652-6656.1995 7592446 PMC177521

[44] Trémoulet F , Duché O , Namane A , Martinie B , Labadie J-C . 2002. A proteomic study of Escherichia coli O157: H7 NCTC 12900 cultivated in biofilm or in planktonic growth mode. FEMS Microbiol Lett 215:7–14. doi:10.1111/j.1574-6968.2002.tb11363.x 12393194

[45] van der Leij F , Witholt B . 1995. Strategies for the sustainable production of new biodegradable polyesters in plants: a review. Can J Microbiol 41:222–238. doi:10.1139/m95-191

[46] de Smet MJ , Eggink G , Witholt B , Kingma J , Wynberg H . 1983. Characterization of intracellular inclusions formed by Pseudomonas oleovorans during growth on octane. J Bacteriol 154:870–878. doi:10.1128/jb.154.2.870-878.1983 6841319 PMC217541

[47] Prieto MA , Bühler B , Jung K , Witholt B , Kessler B . 1999. PhaF, a polyhydroxyalkanoate-granule-associated protein of Pseudomonas oleovorans GPo1 involved in the regulatory expression system for pha genes. J Bacteriol 181:858–868. doi:10.1128/JB.181.3.858-868.1999 9922249 PMC93452

[48] Kourmentza C , Ntaikou I , Kornaros M , Lyberatos G . 2009. Production of PHAs from mixed and pure cultures of Pseudomonas sp. using short-chain fatty acids as carbon source under nitrogen limitation. Desalination 248:723–732. doi:10.1016/j.desal.2009.01.010

[49] Ciesielski S , Mozejko J , Przybyłek G . 2010. The influence of nitrogen limitation on mcl-PHA synthesis by two newly isolated strains of Pseudomonas sp. J Ind Microbiol Biotechnol 37:511–520. doi:10.1007/s10295-010-0698-5 20204456

[50] Velu C , Karthikeyan OP , Brinkman DL , Cirés S , Heimann K . 2021. Biomass pre-treatments of the N_2_-Fixing cyanobacterium Tolypothrix for co-production of methane. Chemosphere 283:131246. doi:10.1016/j.chemosphere.2021.131246 34470734

[51] Kelly S 2022. Proteomics approaches for the analysis of polyhydroxyalkanote production in Pseudomonas putida KT2440. University College Dublin.

