# Supplementary figures and images for: Spatial organization and proteome of a dual-species cyanobacterial biofilm alter among N2-fixing and non-fixing conditions
Source: mSystems. 2023 Jun 7;8(3):e00302-23. doi: 10.1128/msystems.00302-23 (PMC10308936; doi:10.1128/msystems.00302-23)

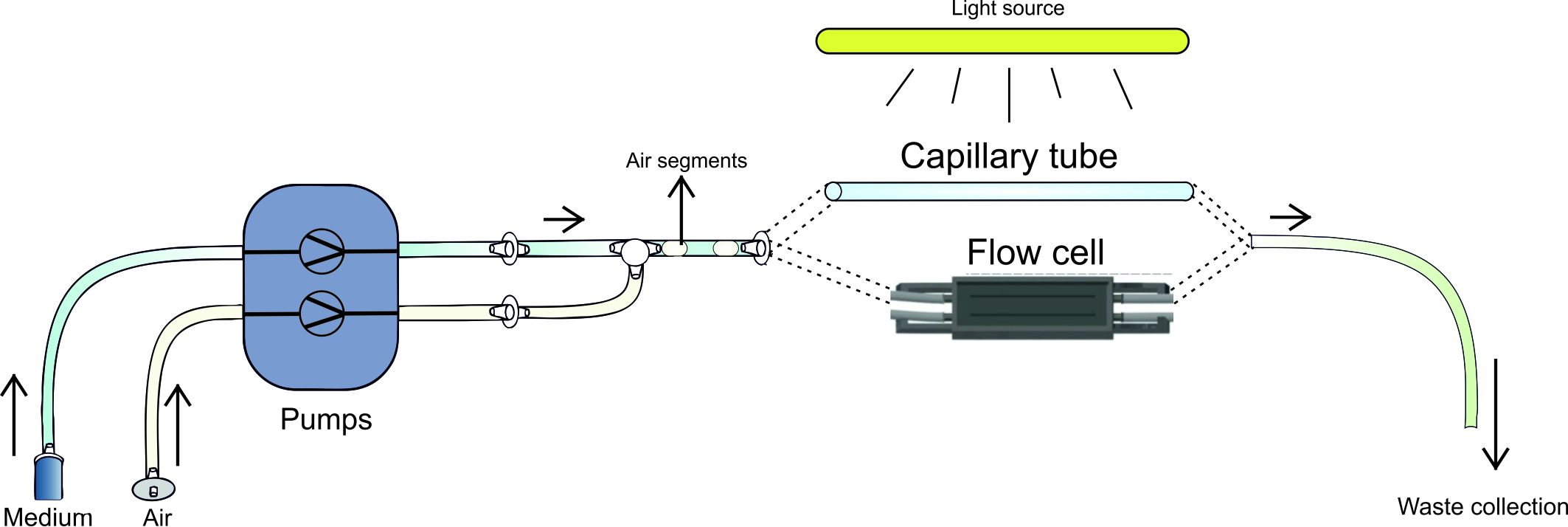

Supplement: Fig. S1 — Illustration of CBR and flow cell system (modified from Bozan et al. (2022)). They both have the same system except the cultivation chamber. [file msystems.00302-23-s0001.tif]

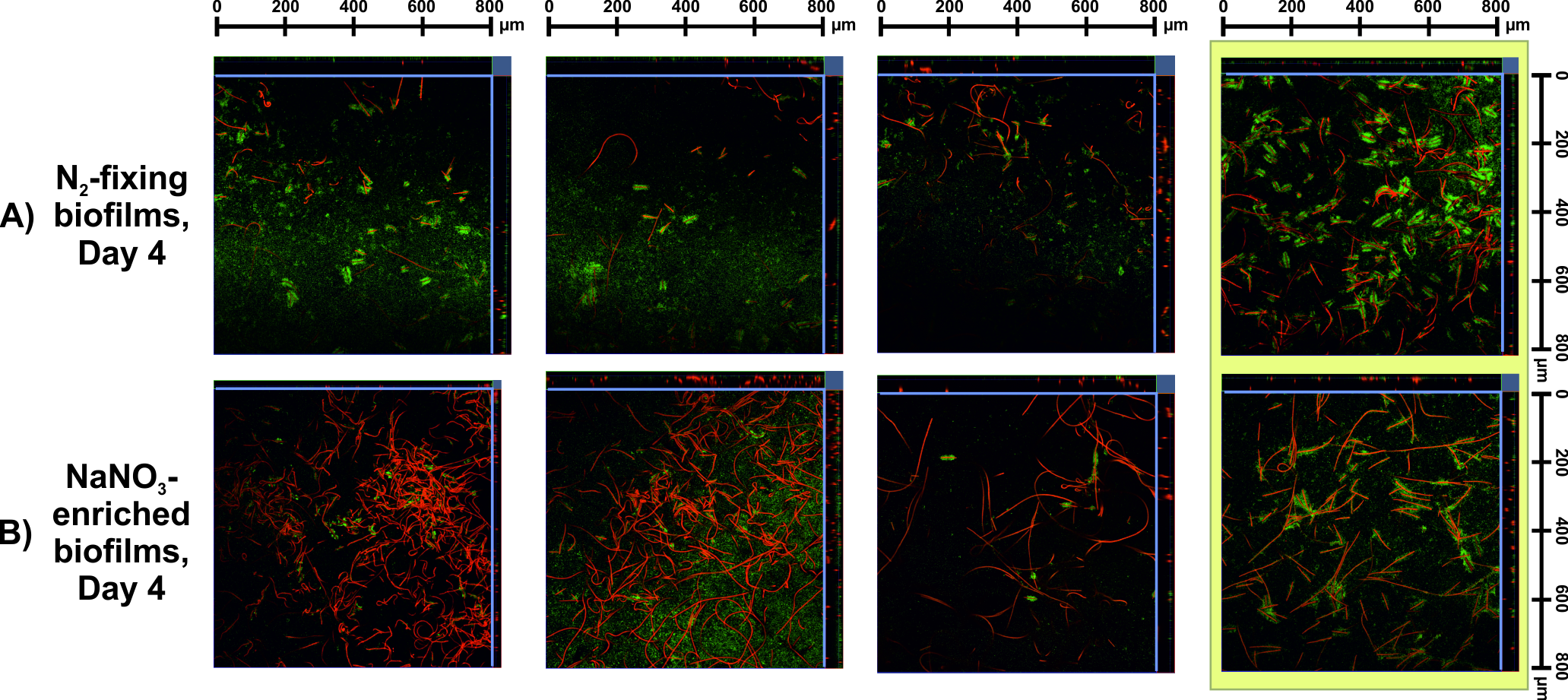

Supplement: Fig. S2 — Additional CLSM images show the co-localization of species in different locations. (A) shows N2-fixing biofilms on day 4, (B) indicates NaNO3-fed biofilms. Images indicated with the yellow box were taken in a different experiment independent from the current study but they were also same conditions. [file msystems.00302-23-s0002.tif]
