# Supplementary material for: Spatial organization and proteome of a dual-species cyanobacterial biofilm alter among N2-fixing and non-fixing conditions
Source: mSystems. 2023 Jun 7;8(3):e00302-23. doi: 10.1128/msystems.00302-23 (PMC10308936; doi:10.1128/msystems.00302-23)
Supplement: Table S1 — Media solutions and their ingredients. [file msystems.00302-23-s0003.docx]

| **Solution type** | **Ingredients** | **Final concentration in media** | **Amount in 1 L** | **100x stock solution** |
| --- | --- | --- | --- | --- |
| **Solution 1A** | NaCl | 17.5 mM | 1.02 g | 102 g / L |
|  | MgSO_4_ x 7.H_2_O | 0.3 mM | 0.074 g | 7.4 g / L |
| **Solution 1B** | NaNO_3_ | 17.5 mM | 1.49 g | 149 g / L |
|  | MgSO_4_ x 7.H_2_O | 0.3 mM | 0.074 g | 7.4 g / L |
| **Solution 2** | K_2_HPO_4_ | 0.175 mM | 0.0305 g | 15.25 g / L |
|  | Na_2_CO_3_ | 0.18 mM | 0.019 g | 9.5 g / L |
| **Trace elements** | CaCl_2_ x 2.H_2_O | 245 μM | 36 mg | 3600 mg / L |
|  | H_3_BO_3_ | 45 μM | 2.78 mg | 278 mg / L |
|  | MnCl_2_ x 4.H_2_O | 9 μM | 1.78 mg | 178 mg / L |
|  | ZnSO_4_ x 7.H_2_O | 0.7 μM | 0.2 mg | 20 mg / L |
|  | Na_2_MoO_4_ x 2.H_2_O | 1.6 μM | 0.39 mg | 39 mg / L |
|  | CuSO_4_ x 5.H_2_O | 0.3 μM | 0.07 mg | 7 mg / L |
|  | Co(NO_3_)_2_ x 6.H_2_O | 0.16 μM | 0.05 mg | 5 mg / L |
|  | FeCl_3_ x 6.H2O/ | 6 μM | 1.62 mg | 162 mg / L |
|  | Na_2_EDTA x 2.H_2_O | 16 µM | 5.95 mg | 595 mg/L |
| **HEPES buffer** | HEPES | 10 mM | 2.38 g | 238.31 g / L |
