# Supplementary material for: Spatial organization and proteome of a dual-species cyanobacterial biofilm alter among N2-fixing and non-fixing conditions
Source: mSystems. 2023 Jun 7;8(3):e00302-23. doi: 10.1128/msystems.00302-23 (PMC10308936; doi:10.1128/msystems.00302-23)
Supplement: Table S2 — Ps_egfp media solutions. [file msystems.00302-23-s0004.docx]

|  | **Ingredients** | **Amount** |
| --- | --- | --- |
| **LB complex medium** | NaCl | 10 g / L |
|  | Tryptone | 10 g / L |
|  | Yeast extract | 5 g / L |
|  | Agar | 15 g / L |
| **M9 medium** | 10 x M9 salt solution | 100 ml |
|  | 1 M MgSO_4_ solution | 2.0 ml |
|  | US* Trace element solution | 1.0 ml |
|  | 20 % (w / v) D(+)-Glucose solution | 25.0 ml |
| **10 x M9 salt solution** | Na_2_HPO_4_ x 7.H_2_O | 128 g / L |
|  | KH_2_PO4 | 30 g / L |
|  | NaCl | 5 g / L |
|  | NH_4_Cl | 10 g / L |
| **US* Trace elements (1000 x)** | 37% fuming HCl | 82.81 ml |
|  | FeSO_4_ x 7.H_2_O | 4.87 g / L |
|  | CaCl_2_ x 2.H_2_O | 4.12 g / L |
|  | MnCl_2_ x 4.H_2_O | 1.50 g / L |
|  | ZnSO_4_ x 7.H_2_O | 1.87 g / L |
|  | H_3_BO_3_ | 0.30 g / L |
|  | Na_2_MoO_4_ x 2.H_2_O | 0.25 g / L |
|  | CuCl_2_ x 2.H_2_O | 0.15 g / L |
|  | Na_2_EDTA x 2.H_2_O | 0.84 g / L |
