# Supplementary material for: Spatial organization and proteome of a dual-species cyanobacterial biofilm alter among N2-fixing and non-fixing conditions
Source: mSystems. 2023 Jun 7;8(3):e00302-23. doi: 10.1128/msystems.00302-23 (PMC10308936; doi:10.1128/msystems.00302-23)
Supplement: Table S3 — Colony counts for determining numbers of Pseudomonas. Number of Ps_egfp colonies in the biofilm and outflow at day 29 together with their ratios. M1 and M2 represent biological replicates of each condition; 'w/o N' represents the N2-fixing, and 'w/ N' represents non-fixing biofilms. [file msystems.00302-23-s0005.docx]

| **Sample type** | ***Ps_egfp* in the biofilm**  **(*Ps_egfp*_biofilm,_ CFU/L)** | ***Ps_egfp* in the outflow**  **(*Ps_egfp*_outflow,_ CFU/L)** | **Ratio of mean values (*Ps_egfp*_biofilm_/ *Ps_egfp*_outflow_)** | **Average ratios**  **(*Ps_egfp*_biofilm_/ *Ps_egfp*_outflow_)** |
| --- | --- | --- | --- | --- |
| M1 w/o N | 1.93 x 10^11^ ± 1.23 x 10^9^ | 2.96 x 10^8^ ± 3.68 x 10^7^ | 652 | 483 ± 238 |
| M2 w/o N | 9.08 x 10^10^ ± 2.72 x 10^10^ | 2.88 x 10^8^ ± 5.23 x 10^7^ | 315 |  |
| M1 w/ N | 4.12 x 10^11^ ± 2.96 x 10^10^ | 1.38 x 10^9^ ± 3.96 x 10^8^ | 299 | 202 ± 136 |
| M2 w/ N | 2.84 x 10^11^ ± 1.23 x 10^9^ | 2.68 x 10^9^ ± 1.34 x 10^8^ | 106 |  |
