# Supplementary material for: Spatial organization and proteome of a dual-species cyanobacterial biofilm alter among N2-fixing and non-fixing conditions
Source: mSystems. 2023 Jun 7;8(3):e00302-23. doi: 10.1128/msystems.00302-23 (PMC10308936; doi:10.1128/msystems.00302-23)
Supplement: Table S4 — Gas composition of CBRs containing Tolypothrix sp. PCC 7712 and Ps_egfp after 13 days of cultivation. Observations were made using CBRs containing Tolypothrix sp. PCC 7712 and Ps_egfp in nitrate-fed and N2-fixing conditions after 13 days of cultivation to demonstrate that there were no anaerobic conditions present throughout biofilm cultivation, where Nitrate could have been assimilated by Ps_egfp. Table S4 illustrates the gas measurement conducted via GC (method description described in Bozan et al., 2022). [file msystems.00302-23-s0006.docx]

|  | Hydrogen | | oxygen | | Nitrogen | |
| --- | --- | --- | --- | --- | --- | --- |
|  | Retention (min) | Amount (%) | Retention (min) | Amount (%) | Retention (min) | Amount (%) |
| M1 w/o N | 1.505 | 0.13 | 1.918 | 26.93 | 2.527 | 72.84 |
| M2 w/o N | 1.493 | 0.05 | 1.917 | 26.75 | 2.523 | 73.21 |
| Air | ND | ND | 1.867 | 25.97 | 2.477 | 74.03 |
| M1 w/ N | n.a. | n.a. | 1.930 | 26.79 | 2.537 | 73.21 |
| M2 w/ N | 1.498 | 0.03 | 1.932 | 26.91 | 2.542 | 73.06 |
| Air | ND | ND | 1.912 | 25.98 | 2.520 | 74.02 |
