# Supplementary material for: Spatial organization and proteome of a dual-species cyanobacterial biofilm alter among N2-fixing and non-fixing conditions
Source: mSystems. 2023 Jun 7;8(3):e00302-23. doi: 10.1128/msystems.00302-23 (PMC10308936; doi:10.1128/msystems.00302-23)
Supplement: Table S5 — T-test results from the comparison of differences between (a) changing depths and (b) NaNO3-fed and N2-fixing biofilms in a certain volume. [file msystems.00302-23-s0007.docx]

a)

|  |  | *Ps_egfp* | | | *Tolypothrix* sp. PCC 7712 | | | Ratios (*Ps_egfp* : *Tolypothrix* sp.) | | |
| --- | --- | --- | --- | --- | --- | --- | --- | --- | --- | --- |
|  | Days | (0-10µm) vs (10-20µm) | (10-20µm) vs (20-30µm) | (0-10µm) vs (20-30µm) | (0-10µm) vs (10-20µm) | (10-20µm) vs (20-30µm) | (0-10µm) vs (20-30µm) | (0-10µm) vs (10-20µm) | (10-20µm) vs (20-30µm) | (0-10µm) vs (20-30µm) |
| N_2_-fed biofilms | 3 | 0.1839 | 0.0994 | 0.0056 | 0.0116 | 0.2474 | 0.1436 | 0.0057 | 0.1060 | 0.0019 |
|  | 4 | 0.3208 | 0.1924 | 0.0269 | 0.0571 | 0.3709 | 0.5716 | 0.0246 | 0.1507 | 0.0009 |
|  | 5 | 0.0007 | 0.0074 | 0.0000 | 0.0163 | 0.5203 | 0.0568 | 0.0000 | 0.0023 | 0.0000 |
|  | 8 | 0.0000 | 0.0001 | 0.0000 | 0.0007 | 0.1069 | 0.0567 | 0.0000 | 0.0001 | 0.0000 |
| NaNO_3_-fed biofilms | 3 | 0.0003 | 0.1131 | 0.0001 | 0.6028 | 0.4959 | 0.8389 | 0.0779 | 0.1686 | 0.0571 |
|  | 4 | 0.5091 | 0.0894 | 0.0144 | 0.3689 | 0.2448 | 0.7651 | 0.0141 | 0.1785 | 0.0004 |
|  | 5 | 0.0078 | 0.0312 | 0.0027 | 0.1490 | 0.0361 | 0.5292 | 0.0000 | 0.2346 | 0.0000 |
|  | 8 | 0.0274 | 0.0315 | 0.0029 | 0.1042 | 0.0774 | 0.9978 | 0.0000 | 0.1300 | 0.0000 |

b)

|  | *Ps_egfp* | | | | *Tolypothrix* sp. | | | | Ratios (*Ps_egfp* : *Tolypothrix* sp.) | | | |
| --- | --- | --- | --- | --- | --- | --- | --- | --- | --- | --- | --- | --- |
|  | (0-10µm) | (10-20µm) | (20-30µm) | (0-30µm) | (0-10µm) | (10-20µm) | (20-30µm) | (0-30 µm) | (0-10µm) | (10-20µm) | (20-30µm) | (0-30 µm) |
| 3 | 0.0195 | 0.0303 | 0.2781 | 0.0362 | 0.0896 | 0.2195 | 0.2920 | 0.1828 | 0.0233 | 0.0153 | 0.2419 | 0.0363 |
| 4 | 0.2844 | 0.7242 | 0.8093 | 0.4643 | 0.0953 | 0.3104 | 0.7218 | 0.2944 | 0.0159 | 0.2930 | 0.9251 | 0.1166 |
| 5 | 0.0045 | 0.0106 | 0.0532 | 0.0013 | 0.0126 | 0.1171 | 0.6904 | 0.0866 | 0.0000 | 0.0010 | 0.0041 | 0.0016 |
| 8 | 0.0097 | 0.0200 | 0.0678 | 0.0018 | 0.0486 | 0.0419 | 0.0750 | 0.0371 | 0.0000 | 0.0000 | 0.0356 | 0.0003 |
