# Supplementary material for: Spatial organization and proteome of a dual-species cyanobacterial biofilm alter among N2-fixing and non-fixing conditions
Source: mSystems. 2023 Jun 7;8(3):e00302-23. doi: 10.1128/msystems.00302-23 (PMC10308936; doi:10.1128/msystems.00302-23)
Supplement: Medium preparation procedure — Preparation procedure for the media used in the study. [file msystems.00302-23-s0009.docx]

**SUPPLEMENTARY DOC FILE**

**1. *Tolypothrix* sp. PCC 7712 medium preparation (BG11-0 and BG11)**

Prepare solutions indicated in **Table S1** as described below:

- Dissolve ‘Solution 1A’ components in dH_2_O and apply filter sterilization.
- Dissolve ‘Solution 1B’ components in dH_2_O and apply filter sterilization.
- Dissolve ‘Solution 2’ components in dH_2_O and apply filter sterilization.
- Dissolve ‘Trace elements’ components in dH_2_O and apply filter sterilization.
- Dissolve ‘HEPES buffer’ in 850 ml solution and adjust **pH 8.2** with NaOH granulates. Complete the solution to 1 L and autoclave or filter sterilize.

(a) Autoclave 968 ml dH_2_O for BG11-0; add each solution shown in **Table S1** in a sterile environment:

- 10 ml of ‘Solution 1A’ (NaCl +MgSO4x 7H2O)
- 2 ml of ‘Solution 2’ (K2HPO4 +Na2CO3)
- 10 ml of ‘Trace Elements’ Solution
- 10 ml of ‘HEPES Buffer’ (1 M HEPES pH 8,2)

(b) Autoclave 968 ml dH_2_O for BG11; add ‘Solution 1B’ instead of ‘Solution 1A’ (**Table S1**) and the rest of the solutions described in section (a).

(c) For BG11-0 and BG11 agar plates:

- 400 ml 1.5% agar sterilized by autoclaving (6 g Bacto Agar (Becton Dickinson)) in 400 ml dH_2_O),
- Add 0.8x sterile solutions as described in (a) and (b) when the agar solution is around 50-60°C,
  - Add 8 ml sterile Na_2_S_2_O_3_ (30%) (1.5 g in 50 ml ddH_2_O).
- Fill the mixture up to 800 ml with ddH_2_0 and distribute the final mixture to Petri dishes (TC Dish100 Standard) by adjusting the final volume to 25 ml for each plate.

**2. *Ps_egfp* medium preparation (BG11-0 and BG11)**

(a) LB complex medium:

- Dissolve components shown in **Table S2** in 800 ml dH_2_0 and fill up to 1000 ml with dH_2_0.
- Autoclave the final mixture for sterilization.

(b) M9 minimal medium:

- Sterilize 1 M MgSO_4_ and 20 % (w / v) D-glucose solutions (**Table S2**) with filter sterilization.
- Add table components to 872 ml ddH_2_O in the given order.

(c) 10 x M9 salts (**Table S2**):

- Adjust ph to 7,2 with 10 M NaOH,
- Autoclave the solution.

(d) US* Trace elements (1000 x):

- Mix chemicals shown in **Table S2** and then filter sterilize the solution.
